# TORCH infections at the maternal–fetal placental transmission: an overview of multi-omics, pathogenesis and innate immune defense

**DOI:** 10.3389/fcimb.2026.1817189

**Published:** 2026-06-08

**Authors:** Sanjeev Nirala, Chunming Huang, Qingchun Mu

**Affiliations:** Affiliated Gaozhou People’s Hospital, Guangdong Medical University, Maoming, China

**Keywords:** decidual natural killer cells, host-pathogen interaction, placental immunology, proteomics, TORCH infections, vertical transmission, viral teratogenesis

## Abstract

The maternal-fetal interface represents a dynamic immunological frontier where pregnancy outcomes are determined by the delicate balance between host defense and microbial pathogenesis. Vertical transmission of pathogens across the placental barrier can lead to devastating consequences including fetal loss, stillbirth, prematurity, and congenital anomalies. The classic TORCH acronym encompasses Toxoplasma gondii, Other agents, Rubella virus, Cytomegalovirus, and Herpes simplex virus, though emerging viruses including Zika virus and SARS-CoV-2 have expanded this spectrum. This review synthesizes current understanding of molecular mechanisms underlying vertical transmission, emphasizing the placenta’s multi-layered defense system encompassing the syncytiotrophoblast physical barrier, specialized decidual immune cell populations including decidual natural killer cells and macrophages, and constitutive antimicrobial signaling pathways focusing on the placenta’s multi-layered defense system encompassing the syncytiotrophoblast physical barrier, specialized decidual immune cell populations including decidual natural killer cells and macrophages, and constitutive antimicrobial signaling pathways. Concurrently, we examine pathogen strategies to subvert these defenses through receptor manipulation, immune evasion, and intracellular replication niches. A major focus is dedicated to the impact of proteomics and single-cell multi-omics in deconvoluting the host-pathogen interactome and identifying biomarkers of fetal injury. Recent seroprevalence studies demonstrate that younger women represent the most susceptible population to acute TORCH infections, highlighting the need for targeted screening programs. This synthesis provides a framework for developing precision diagnostics and targeted interventions to prevent vertical transmission and reduce the global burden of congenital infections.

## 1 Introduction

### 1.1 The global burden of congenital infections

Congenital infections remain a leading cause of fetal and neonatal morbidity, mortality, and long-term neurodevelopmental disability worldwide (Parums, 2024; Megli and Coyne, 2022). Vertical transmission—the translocation of pathogens across the placental barrier from mother to fetus—can precipitate a devastating spectrum of outcomes, including structural birth defects, severe growth restriction, spontaneous abortion, stillbirth, and preterm delivery (Parums, 2024; Megli and Coyne, 2022). The placental maternal-fetal interface provides physical, molecular, and immunological mechanisms that successfully protect the developing fetus from most pathogens, yet select microorganisms have evolved strategies to circumvent these defenses (Racicot and Mor, 2017).

The term “TORCH” was coined to group pathogens with similar clinical presentations in neonates, including Toxoplasma gondii, rubella virus, cytomegalovirus (CMV), and herpes simplex virus (HSV), with the “O” representing other agents such as syphilis, varicella-zoster virus, parvovirus B19, and emerging threats including Zika virus (ZIKV) (Coyne and Lazear, 2016). Recent seroprevalence studies in childbearing age women have demonstrated that rubella vaccination programs have achieved high immunological protection (87%), followed by CMV (79.5%), while susceptibility to Toxoplasma gondii (85.1%), HSV-1 (69%), and HSV-2 (78.5%) remains significant, with younger women (16–25 years) representing the most susceptible population (Qin et al., 2021). The maternal-fetal interface represents a dynamic immunological frontier where pregnancy outcomes are determined by the delicate balance between host defense and microbial pathogenesis. Vertical transmission of pathogens across the placental barrier can lead to devastating consequences including fetal loss, stillbirth, prematurity, and congenital anomalies (Parums, 2024). The classic TORCH acronym (Toxoplasma gondii, Other agents, Rubella virus, Cytomegalovirus, Herpes simplex virus) encompasses historically important pathogens, though emerging viruses including Zika virus (ZIKV) and SARS-CoV-2 have expanded this spectrum (Megli and Coyne, 2022). This comprehensive review synthesizes current understanding of the molecular mechanisms underlying vertical transmission, focusing on the placenta’s multi-layered defense system encompassing the syncytiotrophoblast physical barrier, specialized decidual immune cell populations including decidual natural killer (dNK) cells and macrophages, and constitutive antimicrobial signaling pathways (Espino et al., 2021). Concurrently, we examine pathogen strategies to subvert these defenses through receptor manipulation, immune evasion, and intracellular replication niches. A major focus is dedicated to the impact of proteomics and single-cell multi-omics in deconvoluting the host-pathogen interactome and identifying biomarkers of fetal injury (Piekos et al., 2025). Recent seroprevalence studies demonstrate that younger women (16–25 years) represent the most susceptible population to acute TORCH infections, highlighting the need for targeted screening programs (Qin et al., 2021). This synthesis provides a framework for developing precision diagnostics and targeted interventions to prevent vertical transmission and reduce the global burden of congenital infections.

The term “TORCH” was coined to group pathogens with similar clinical presentations in neonates, including Toxoplasma gondii, rubella virus, cytomegalovirus (CMV), and herpes simplex virus (HSV), with the “O” representing other agents such as syphilis (Treponema pallidum), varicella-zoster virus (VZV), parvovirus B19, and emerging threats including Zika virus (ZIKV) and SARS-CoV-2 (Coyne and Lazear, 2016; Parums, 2024; Megli and Coyne, 2022).

### 1.2 Contemporary understanding of pathogenesis

Contemporary research demonstrates that pathogenic sequelae result from a complex triad: specific pathogen virulence determinants, gestational timing of maternal infection, and the unique immune environment of the maternal-fetal interface (Racicot and Mor, 2017; Ander et al., 2019). Infection during organogenesis (first trimester) carries the highest risk for major structural anomalies, whereas later infections may cause isolated sensory deficits or growth restriction (Girsch et al., 2022). The molecular mechanisms underlying these outcomes involve direct cytopathic damage to fetal progenitor cells, dysregulation of placental angiogenesis and nutrient transport, and immunopathology driven by dysregulated inflammatory responses (Megli and Coyne, 2022).

### 1.3 Scope and organization

This comprehensive review synthesizes current literature from 2015-2025, with emphasis on: (1) architectural and immunological foundations of placental defense; (2) molecular pathogenesis of TORCH pathogens; (3) emerging infectious threats; (4) multi-omics technologies in placental infection research; and (5) translational implications for diagnosis and therapy.

### 1.4 The placenta as a dynamic immunological processor

The maternal-fetal interface functions not as a passive static barrier but as a dynamic, stage-specific immunological processor that continuously integrates spatial, temporal, and immunological information to balance the competing demands of fetal tolerance and antimicrobial defense (Megli and Coyne, 2022; Ander et al., 2019). This framework rests on four core principles. First, spatial specialization dictates that distinct placental compartments exhibit differential susceptibility to pathogens: the syncytiotrophoblast, with its constitutive type III interferon production and cortical actin network, serves as the primary physical and antiviral barrier (Bayer et al., 2016; Zeldovich et al., 2013), while extravillous trophoblasts and Hofbauer cells, despite their critical roles in placental development, represent vulnerability points that pathogens have evolved to exploit (Tabata et al., 2016; Quicke et al., 2016). Second, gestational timing fundamentally alters this defense landscape, with first-trimester infections posing the highest risk for structural anomalies due to ongoing organogenesis, limited trophoblast fusion, and an evolving immune cell repertoire, whereas later infections may trigger inflammatory cascades leading to preterm birth or growth restriction (Racicot and Mor, 2017; Girsch et al., 2022). Third, the placenta operates under an inherent tolerance-defense trade-off: mechanisms that prevent rejection of the semi-allogeneic fetus—such as regulatory T cell expansion, decidual NK cell functional polarization away from cytotoxicity, and M2-skewed macrophage phenotypes—can simultaneously create permissive niches for intracellular pathogens that subvert these immunoregulatory pathways (Moffett and Colucci, 2014; Rowe et al., 2012; Thomas et al., 2021). Fourth, multi-omics technologies have been instrumental in deconvoluting these dynamics, revealing that what was once described as “immune privilege” is better understood as “immunologically distinct and tightly regulated” (Vento-Tormo et al., 2018; Piekos et al., 2025). Throughout this review, the authors return to this framework to demonstrate how specific pathogens exploit vulnerabilities at each level and how emerging technologies are revealing novel intervention points. The Conclusion revisits this framework to synthesize translational implications.

## 2 Architectural and immunological foundations of placental defense

**Figure 1:**
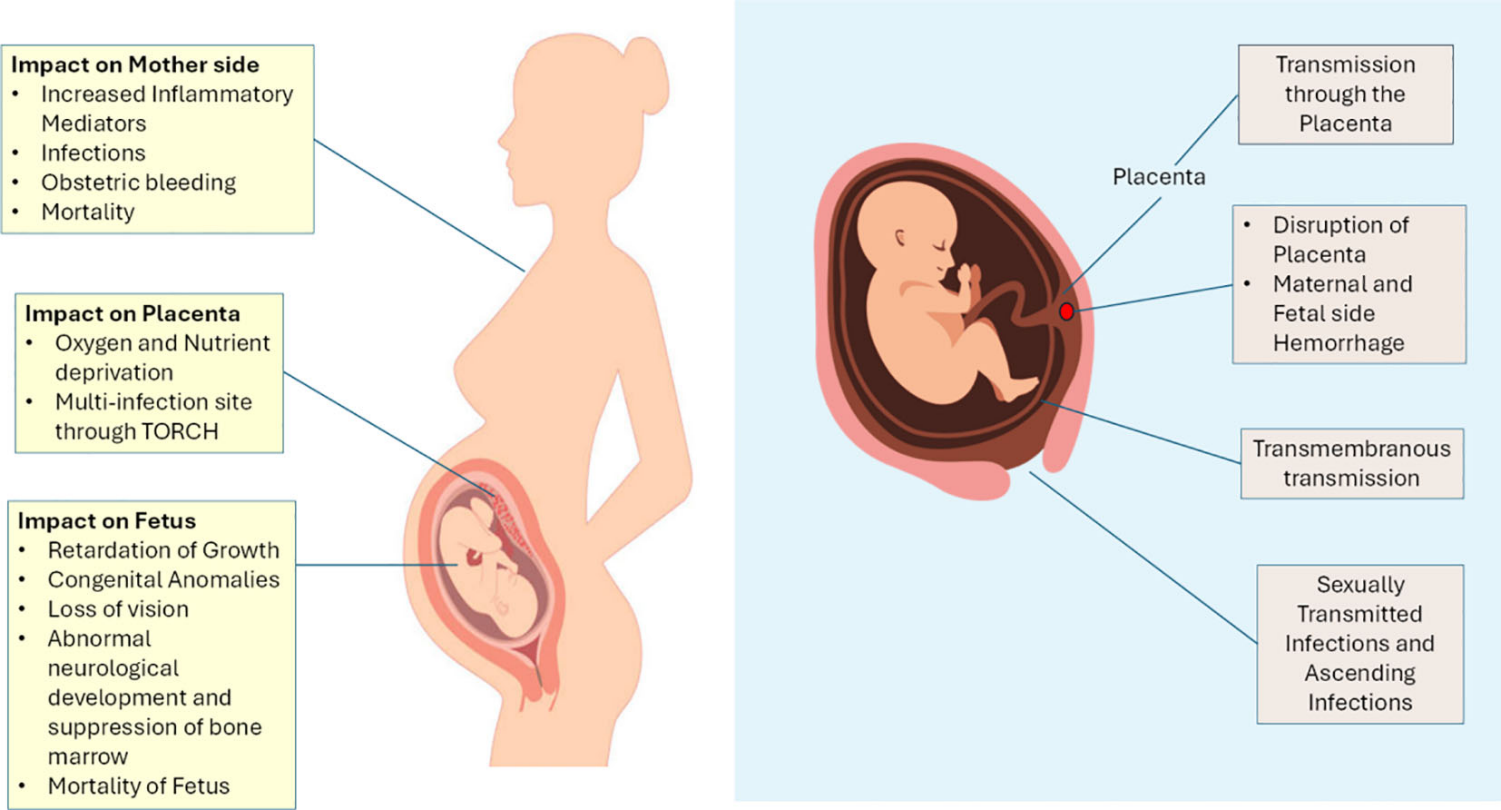
Principal mechanisms for pathogenic invasion of the intra-amniotic space. Key routes include direct hematogenous transplacetion of the decidual–placental interface; and transmission via fetal–maternal hemorrhage. A distinct, non-hematogenous pathway is ascending infection from the lower genital tract, whereby microorganisms colonize and traverse the chorioamniotic membranes. The sequelae of such infntal transmission, where pathogens circulating in the maternal blood cross the syncytiotrophoblast barrier; invasion secondary to placental damage or disrupections are tripartite, with potential for maternal morbidity, direct fetal injury (including developmental anomalies and growth restriction), and placental pathology (e.g., villitis or funisitis), largely mediated by the interplay between direct microbial cytopathy and a dysregulated maternal–fetal inflammatory response. This image illustrates the conceptual distinction between hematogenous transmission (direct transplacental spread) and ascending infection, emphasizing that different pathogens exploit distinct routes requiring different intervention strategies (Racicot and Mor, 2017; Megli and Coyne, 2022).

**Figure 2:**
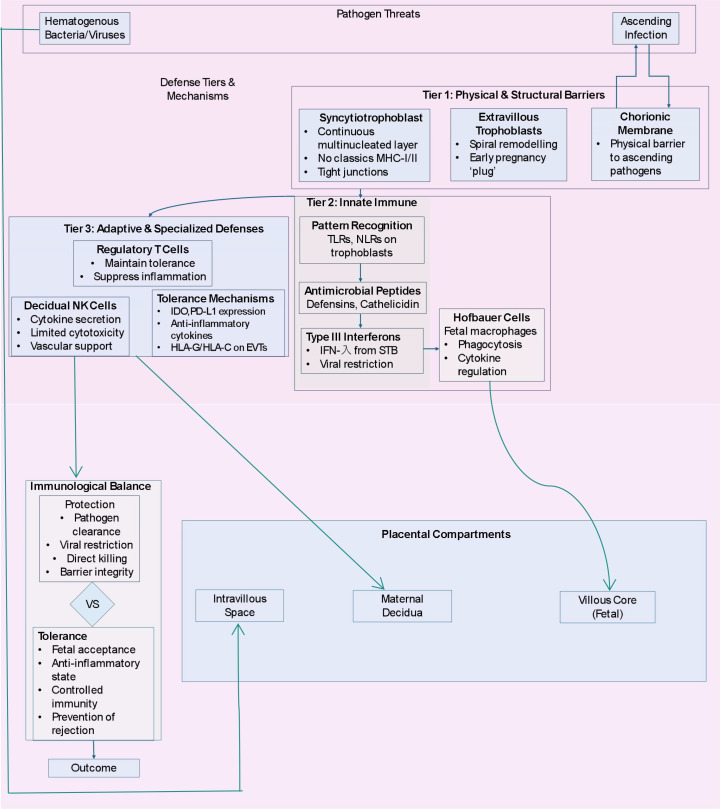
Placental defenses against pathogens: The placenta is not a passive barrier but an active immunological organ that orchestrates a sophisticated, multi-layered defense strategy to protect the developing fetus from pathogens while maintaining immune tolerance to the semi-allogeneic fetus. This defense system balances protection and tolerance, preventing harmful immune overreactions that could trigger preterm labor or fetal rejection. Two defenses mainly 1.Physical and Structural Barriers: Syncytiotrophoblast Layer: This continuous, multinucleated cell layer covering the chorionic villi forms the primary cellular barrier between maternal blood and fetal tissues. It lacks classic surface MHC class I and II molecules, reducing recognition by maternal T cells. Its tight intercellular connections limit pathogen entry. Extravillous Trophoblasts (EVTs): These invasive cells remodel maternal spiral arteries and form a cytotrophoblast plug in early pregnancy, limiting blood flow and pathogen access to the intervillous space. Chorionic Membrane: An additional physical and immunological barrier against ascending infections from the lower genital tract. 2. Innate Immune Defenses (First Line of Response): The placental cells express a wide array of Pattern Recognition Receptors (PRRs), such as Toll-like receptors (TLRs), which detect conserved pathogen molecules. Antimicrobial Peptides (AMPs): Trophoblasts and other placental cells produce AMPs, for example defensins and cathelicidin, that can directly disrupt bacterial, viral, and fungal membranes. Type III Interferons (IFN-λ): The syncytiotrophoblast preferentially produces IFN-λ, which establishes an antiviral state in placental cells without triggering the more inflammatory responses associated with Type I interferons. Hofbauer Cells: These are fetal-derived macrophages residing in the villous stroma. They act as phagocytic cells, clearing debris and pathogens, and help regulate the local immune environment through cytokine secretion. This image synthesizes the multi-layered defense framework, demonstrating that physical barriers (syncytiotrophoblast) and innate immune mechanisms (IFN-λ, antimicrobial peptides, inflammasome priming) operate in parallel to create overlapping protection (Bayer et al., 2016; Gomez-Lopez et al., 2019; Zeldovich et al., 2013).

The human placenta functions not as a passive filter but as a highly active immunological organ orchestrating multi-tiered defense strategies while maintaining tolerance to the semi-allogeneic fetus (Megli and Coyne, 2022; Ander et al., 2019; Espino et al., 2021) as illustrated in **Table 1**; **Figures 1**, **2**.

**Table 1:** Immune cell populations at the maternal-fetal interface.

Cell type	Abundance (1st trimester)	Surface markers	Primary functions	Role in infection	Key references
Decidual NK Cells	~70% of decidual leukocytes	CD56bright, CD16-, CD9+, KIR+	Spiral artery remodeling, trophoblast invasion promotion	Granulysin transfer to infected trophoblasts; limited cytotoxicity	Vento-Tormo et al., 2018; Huhn et al., 2020; Moffett and Colucci, 2014
Decidual Macrophages	~20-25%	CD206+, CD163+, IL-10hi, CD68+	Tissue remodeling, apoptotic debris clearance	Phagocytosis; potential pathogen reservoir for HIV, ZIKV	Thomas et al., 2021; Quicke et al., 2016
Hofbauer Cells (Fetal Macrophages)	Variable (gestational age-dependent)	CD206+, CD163+, CD68+, CD14+	Villous development, immune regulation, angiogenesis	Phagocytic sentinel; Zika virus replication niche	Reyes and Golos, 2018; Quicke et al., 2016
Regulatory T Cells	~5-10%	CD4+, CD25+, FOXP3+, CTLA-4+	Fetal tolerance maintenance, immune modulation	Suppress anti-fetal immunity; modulate inflammatory responses	Buchholz and Flossdorf, 2018; Rowe et al., 2012
Innate Lymphoid Cells	Low (increasing in early pregnancy)	ILC1 (T-bet+), ILC2 (GATA-3+), ILC3 (RORγt+)	Early cytokine production, tissue homeostasis	Antigen-independent defense; rapid response to infection	Huhn et al., 2020
γδ T Cells	Low	TCRγδ+, CD8+/CD8-, Vδ1+ predominance	Cytotoxicity, cytokine production, antigen presentation	First-line defense; recognize stress signals without MHC restriction	Tilburgs et al., 2008
Dendritic Cells	<1%	CD11c+, HLA-DR+, CD1c+, CD141+	Antigen presentation, T cell priming	Bridge innate and adaptive immunity; tolerance induction	Vento-Tormo et al., 2018

### 2.1 Structural and biomechanical barriers

The hemochorial structure places fetal chorionic villi in direct contact with maternal blood. The syncytiotrophoblast (STB), a continuous multinucleated syncytium formed by fusion of underlying cytotrophoblasts, constitutes the primary cellular barrier (Turco and Moffett, 2019). The STB’s resilience is underpinned by a dense submembranous cortical actin cytoskeleton and the absence of traditional tight junctions, rendering it resistant to paracellular migration used by many bacteria and parasites (Zeldovich et al., 2013; Robbins and Bakardjiev, 2012). This physical defense is complemented by the chorionic membrane and cervical mucus plug, which guard against ascending infection (Ibarra et al., 2025). Advanced microfluidic “placental chip” models now enable real-time study of these barrier functions under physiological flow conditions (Gonçalves et al., 2025).

### 2.2 Innate immune defense mechanisms

**Figure 3:**
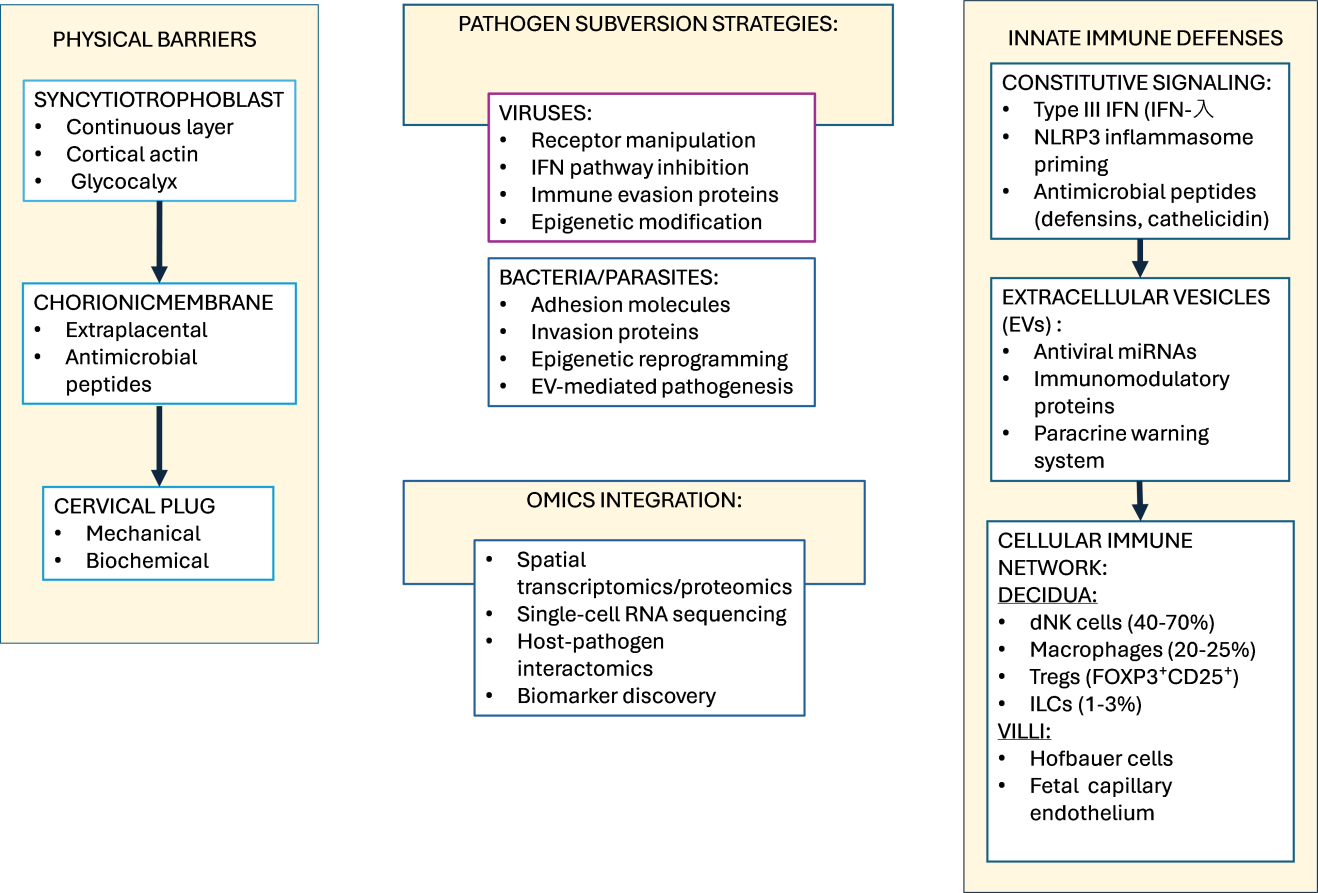
Integrated schematic of physical and immunological placental defenses. This image synthesizes the multi-layered defense framework introduced in Section 2, demonstrating that the placenta employs overlapping and spatially organized defense mechanisms that operate in parallel to create redundant protection against pathogen invasion across the maternal-fetal interface. The left panel depicts the anatomical organization from maternal blood (top) through fetal circulation (bottom), illustrating the syncytiotrophoblast (STB) as the primary cellular barrier whose dense cortical actin cytoskeleton and absence of traditional tight junctions render it resistant to paracellular pathogen migration (Zeldovich et al., 2013; Robbins and Bakardjiev, 2012). The STB constitutively secretes type III interferons (IFN-λ), depicted as small colored spheres, establishing a baseline antiviral state without the pro-inflammatory effects of type I interferons (Bayer et al., 2015; Bayer et al., 2016). Beneath the STB, the cytotrophoblast (CTB) layer and villous stroma contain Hofbauer cells (fetal-derived macrophages), which act as phagocytic sentinels but can paradoxically serve as replication niches for pathogens including Zika virus and HIV (Reyes and Golos, 2018; Quicke et al., 2016; Ojwach et al., 2024). The right panel provides a molecular view of defense mechanisms operating at the syncytiotrophoblast surface, showing how pattern recognition receptors (TLR3, TLR4, TLR7/8, RIG-I, MDA5) trigger NF-κB-mediated production of inflammatory cytokines and IRF-mediated production of type III interferons (Motomura et al., 2023; Koga and Mor, 2010). The figure also illustrates the constitutive NLRP3 inflammasome priming in trophoblasts, leading to low-level production of IL-1β and IL-18 that primes the tissue for rapid response while requiring tight control to avoid preterm labor (Gomez-Lopez et al., 2019). Finally, the release of extracellular vesicles (exosomes) loaded with antiviral miRNAs (including the primate-specific C19MC cluster) is depicted, showing how trophoblasts can transfer viral resistance to neighboring cells and maternal immune cells as a paracrine warning system (Delorme-Axford et al., 2013; Mouillet et al., 2015). The conceptual take-home message is that placental defense is not a single barrier but an integrated system of physical, cellular, and molecular mechanisms that collectively balance fetal tolerance with antimicrobial protection.

The placenta exhibits unique innate immune (**Figure 3**) properties distinct from peripheral tissues (Racicot and Mor, 2017; Ander et al., 2019; Motomura et al., 2023).

#### 2.2.1 Constitutive type III interferon production

Unlike most cells that induce interferons only upon infection, trophoblasts constitutively secrete IFN-λ, establishing a baseline antiviral state (Bayer et al., 2015; Bayer et al., 2016). This signaling is exquisitely regulated in a gestation-dependent manner, peaking during early placentation to protect against viral invasion (Bayer et al., 2016; Harrison and Bailey-Hytholt, 2025). IFN-λ signals through a unique receptor complex (IL-28Rα/IL-10Rβ) expressed on trophoblasts and epithelial cells, inducing hundreds of interferon-stimulated genes (ISGs) that establish an antiviral environment without the pro-inflammatory effects of type I interferons (Johns et al., 2022).

#### 2.2.2 Trophoblast-intrinsic pathogen sensing

Trophoblasts express a broad repertoire of Toll-like receptors (TLRs) and cytosolic sensors (RIG-I, MDA5) (Motomura et al., 2023). TLR4 recognizes bacterial lipopolysaccharide, TLR3 recognizes viral double-stranded RNA, and TLR7/8 recognize single-stranded RNA (Koga and Mor, 2010). Their activation triggers robust production of antimicrobial peptides, chemokines, and cytokines, orchestrating a localized defense without necessarily initiating a systemic maternal inflammatory response (Motomura et al., 2023).

#### 2.2.3 Extracellular vesicle-mediated cross-talk

Trophoblasts constitutively release exosomes and microvesicles loaded with antiviral miRNAs and immunomodulatory proteins (Delorme-Axford et al., 2013; Bayer et al., 2016). These EVs can be taken up by neighboring maternal immune cells or other trophoblasts, acting as a paracrine warning system and defense amplifier (Delorme-Axford et al., 2013). The chromosome 19 miRNA cluster (C19MC), expressed almost exclusively in primate trophoblasts, is packaged into exosomes and confers viral resistance to recipient cells (Delorme-Axford et al., 2013; Mouillet et al., 2015).

#### 2.2.4 Inflammasome activation

The placenta maintains constitutive NLRP3 inflammasome priming, leading to low-level production of IL-1β and IL-18 (Gomez-Lopez et al., 2019). This primes the tissue for rapid response to bacterial insults but requires tight control to avoid excessive inflammation linked to adverse outcomes including preterm labor and preeclampsia (Gomez-Lopez et al., 2019). The NLRP7 inflammasome appears particularly important in trophoblasts, responding to microbial lipopeptides (Abi Abdallah et al., 2026).

### 2.3 Specialized immune cell populations at the interface

**Figure 4:**
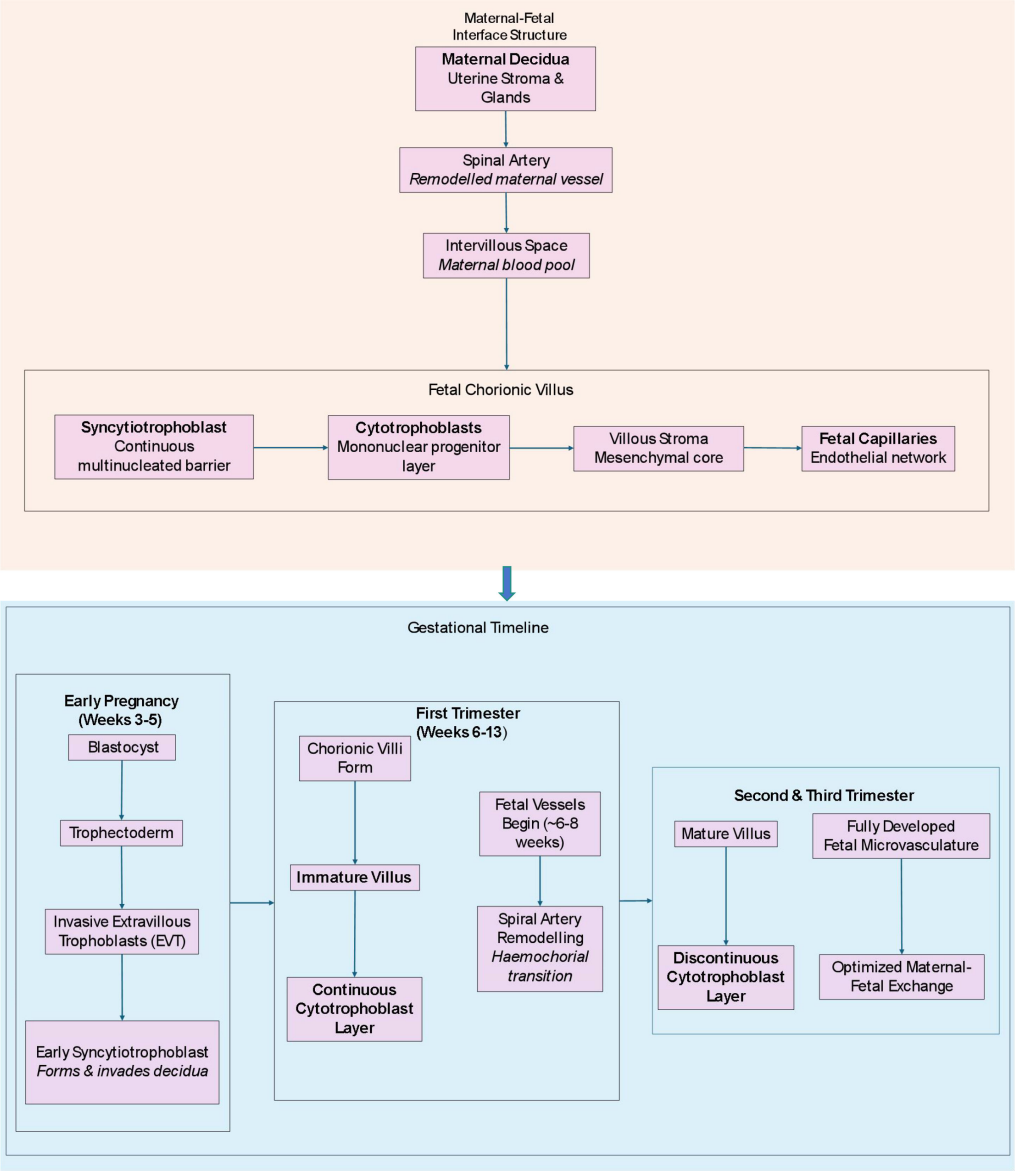
Developmental timeline and cellular architecture of chorionic villi. This image illustrates the dynamic evolution of chorionic villus architecture across gestation and the corresponding changes in cellular composition and immune defense capacity, emphasizing the critical principle that gestational timing fundamentally alters susceptibility to vertical transmission (Racicot and Mor, 2017; Girsch et al., 2022). The top panel presents a developmental timeline from weeks 4–40 of gestation, showing that first-trimester villi (weeks 4-12) are characterized by a thick cytotrophoblast layer, incomplete syncytial fusion, and limited vascularization—features that create relative vulnerability to pathogens such as cytomegalovirus and Toxoplasma gondii during organogenesis (Megli and Coyne, 2022; Tabata, 2019). The middle panel provides cross-sectional schematics of chorionic villi at three critical gestational stages: first trimester (weeks 6-12), second trimester (weeks 13-24), and third trimester (weeks 25-40). In the first trimester, the villous core contains abundant Hofbauer cells (depicted as large round cells with phagocytic vacuoles) and mesenchymal cells, with a discontinuous STB layer that allows cytotrophoblasts direct contact with maternal blood (Turco and Moffett, 2019). By the second trimester, the STB has matured into a continuous multinucleated layer, cytotrophoblast density decreases, and fetal capillaries begin to migrate toward the villous surface, reducing diffusion distance but also creating potential entry points for hematogenously disseminated pathogens (Colson et al., 2021). In the third trimester, the villous membrane thins dramatically (to approximately 3-5 μm), syncytial fusion is complete, and Hofbauer cell numbers decline—changes that optimize nutrient and gas exchange but may alter pathogen susceptibility profiles (Reyes and Golos, 2018). The bottom panel maps the spatial distribution of key immune defense molecules across the villus, showing that IFN-λ production is highest in the STB (depicted as dense stippling), TLR expression is concentrated on the STB apical surface, and antimicrobial peptides (defensins, cathelicidin) are secreted into both the intervillous space and the villous stroma (Bayer et al., 2016; Koga and Mor, 2010). The figure also indicates the differential susceptibility of specific cell populations to TORCH pathogens: extravillous trophoblasts (EVTs) are highly permissive to HCMV via PDGFRα expression (Girsch et al., 2022), Hofbauer cells are preferential targets for ZIKV (Quicke et al., 2016), and syncytiotrophoblast is remarkably resistant to HSV and SARS-CoV-2 due to lack of entry receptors (Dhar et al., 2025; Tang et al., 2024). The conceptual take-home message is that placental architecture and immune capacity are not static but evolve dynamically across gestation, creating a shifting landscape of susceptibility and resistance that explains why first-trimester infections carry the highest risk for congenital anomalies while later infections may cause isolated deficits or preterm birth (Racicot and Mor, 2017; Megli and Coyne, 2022).

Single-cell RNA sequencing (scRNA-seq) and high-dimensional cytometry have revolutionized our understanding of the decidual and placental immune landscape (Vento-Tormo et al., 2018; Suryawanshi et al., 2018; Tang et al., 2024) as illustrated in **Figure 4**.

#### 2.3.1 Decidual natural killer cells

Decidual NK cells are the most abundant leukocyte in early decidua, comprising approximately 70% of decidual leukocytes (Moffett and Colucci, 2014). They are distinct from their peripheral counterparts in phenotype, transcriptional profile, and function (Vento-Tormo et al., 2018). dNK cells are CD56bright CD16- and lack cytotoxic granules, rendering them poorly cytotoxic (Kopcow et al., 2005). Instead, they secrete a unique profile of cytokines and growth factors including GM-CSF, VEGF, and IL-8 that promote spiral artery remodeling and trophoblast invasion (Hanna et al., 2006). Recent studies have demonstrated that dNK cells can directly transfer antimicrobial peptides including granulysin to infected trophoblasts through nanotubules, enabling pathogen clearance without fetal cell death (Espino et al., 2021).

#### 2.3.2 Decidual and Hofbauer macrophages

Decidual macrophages exhibit a predominantly M2-like, immunoregulatory phenotype (CD206+, CD163+, IL-10hi) that supports tissue remodeling and clears apoptotic debris (Thomas et al., 2021). Fetal-derived Hofbauer cells within the villous stroma share this phenotype and are critical for villous development and immune regulation (Reyes and Golos, 2018). Both populations act as phagocytic sentinels but can be hijacked as replication niches by pathogens including Zika virus and HIV (Quicke et al., 2016; Ojwach et al., 2024).

#### 2.3.3 Regulatory T cells (Tregs)

A specialized population of regulatory T cells expands during pregnancy and is essential for maintaining fetal tolerance (Buchholz and Flossdorf, 2018; Rowe et al., 2012). These cells suppress harmful maternal anti-fetal immunity through multiple mechanisms including CTLA-4 engagement, IL-10 production, and TGF-β secretion, while also modulating inflammatory responses to infection to prevent immunopathology (Buchholz and Flossdorf, 2018).

#### 2.3.4 Innate lymphoid cells and γδ T cells

Tissue-resident ILCs and γδ T cells in the decidua provide early, antigen-independent defense through rapid cytokine production and direct cytotoxicity against infected cells, forming a crucial first line of defense (Huhn et al., 2020; Tilburgs et al., 2008).

## 3 Molecular pathogenesis of TORCH pathogens

**Figure 5:**
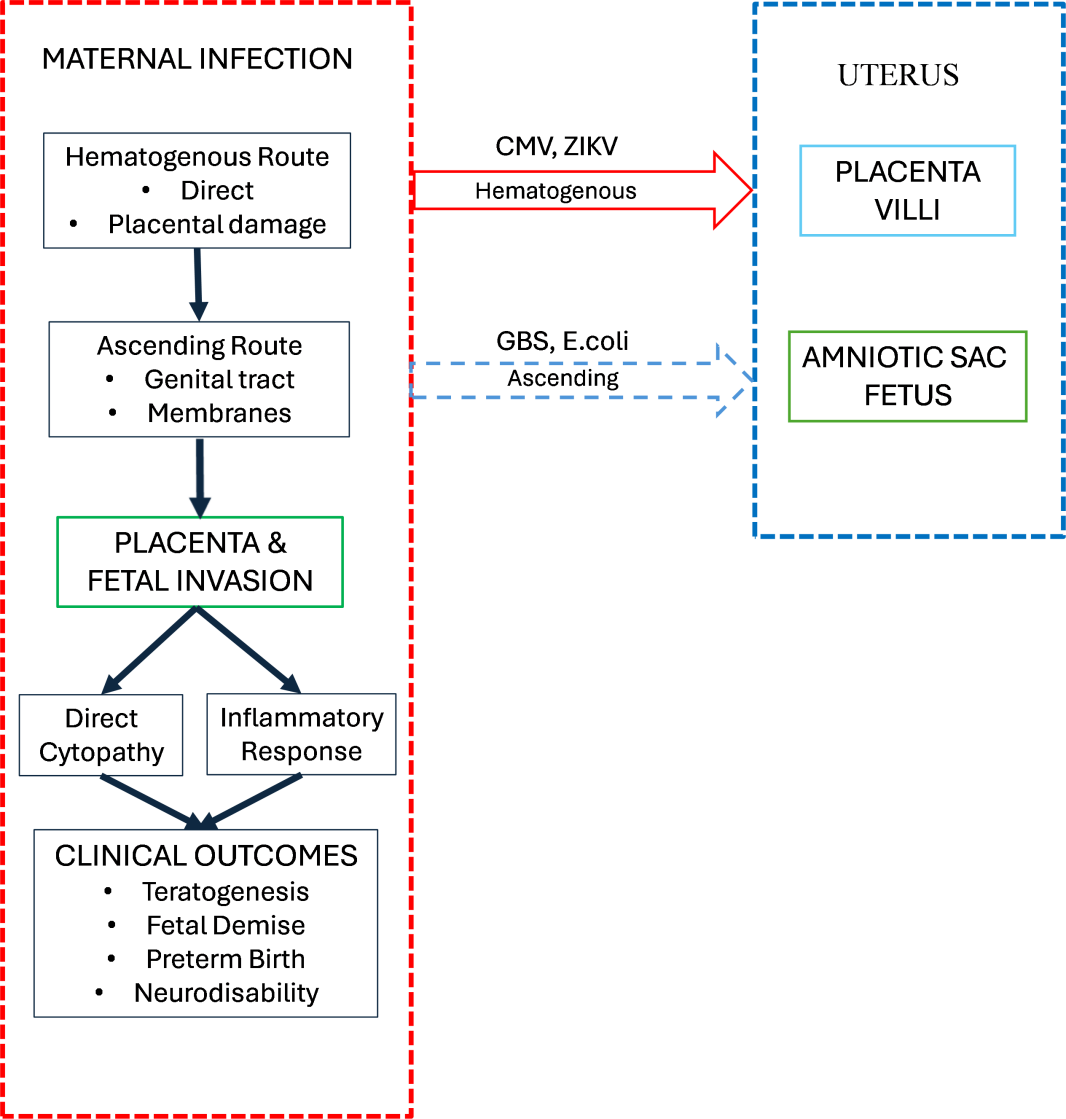
Pathogen transmission routes (hematogenous, ascending) and clinical sequelae. It illustrates that maternal infection is the initiating event in all scenarios (represented by the red box at left). From this maternal infection, pathogens may disseminate to the placenta via two principal routes: (1) hematogenous transmission (top pathway, solid arrows), where pathogens circulating in maternal blood breach the syncytiotrophoblast barrier (Megli and Coyne, 2022); or (2) ascending infection (bottom pathway, dashed arrows), where pathogens colonize the lower genital tract and traverse the chorioamniotic membranes (Ibarra et al., 2025). Once the placenta is colonized (blue box, center), pathogens may either cause placental pathology (e.g., villitis, intervillositis, fibrin deposition) without fetal transmission, or proceed to fetal infection (green box, right) via the umbilical circulation or direct extension (Racicot and Mor, 2017). The arrows from the red box to the blue box therefore indicate “maternal infection → placental colonization, ” not simultaneous infection. For specific pathogens illustrated: CMV and Zika virus (ZIKV) follow the hematogenous route, with maternal viremia preceding placental detection by days to weeks (Girsch et al., 2022; Miner and Diamond, 2017). Listeria monocytogenes can utilize both routes but is shown primarily via hematogenous spread in this schematic (Lecuit et al., 2004; Bakardjiev et al., 2006). The temporal sequence is therefore: (1) maternal infection (red box), (2) placental colonization (blue box), and (3) either placental pathology alone or fetal infection (green box).

This section details the latest molecular insights into how specific pathogens breach placental defenses and cause fetal disease as illustrated in **Table 2** and **Figure 5**.

**Table 2:** TORCH and emerging pathogens: molecular mechanisms and clinical consequences.

Pathogen	Classification	Primary cellular targets	Key virulence mechanisms	Host restriction factors	Congenital effects	Key references
Cytomegalovirus (HCMV)	Herpesvirus (dsDNA)	Extravillous trophoblasts, Hofbauer cells, decidual stromal cells	US28/UL141 (NK cell evasion), pp65 (IRF3 inhibition), US28 (chemokine scavenging), IE1/IE2 (STAT interference)	APOBEC3A (hypermutation), viperin, tetherin	Sensorineural hearing loss (50%), microcephaly, IUGR, hepatosplenomegaly, petechiae	Girsch et al., 2022; Weisblum et al., 2017; Britt, 2017
Zika Virus (ZIKV)	Flavivirus (+ssRNA)	Hofbauer cells, placental fibroblasts, cytotrophoblasts, endothelial cells, neural progenitor cells	NS5-mediated STAT2 degradation, NS1 (endothelial dysfunction), NS4A/4B (autophagy inhibition)	IFITMs, viperin, ISG15	Microcephaly, intracranial calcifications, brain atrophy, arthrogryposis, ocular abnormalities	Grant et al., 2016; Quicke et al., 2016; Miner and Diamond, 2017
Herpes Simplex Virus (HSV-1/2)	Herpesvirus (dsDNA)	Extravillous trophoblasts (STB resistant due to lack of nectin-1/HVEM)	Latency establishment (sensory ganglia), ICP47 (TAP inhibition), vhs (host shutoff)	SAMHD1, APOBEC3 proteins	CNS disease (encephalitis), disseminated infection, skin/eye/mouth lesions, high mortality	Dhar et al., 2025; El-Gharbawy and Vockley, 2018
SARS-CoV-2	Coronavirus (+ssRNA)	Limited (minimal ACE2 expression in trophoblasts); vascular endothelium	TLR4 activation (vascular pathology), spike protein-mediated endothelial dysfunction	Low ACE2/TMPRSS2 expression, constitutive IFN-λ	Rare vertical transmission (<2%); placental insufficiency, stillbirth from placentitis	Tang et al., 2020; Schwartz et al., 2022; Barcelos et al., 2021
Rubella Virus	Togavirus (+ssRNA)	Endothelial cells, placental stroma	p53 inactivation, mitochondrial disruption, cytoskeletal damage	Unknown	Congenital rubella syndrome: cataracts, heart defects, SNHL, IUGR	Parums, 2024
Toxoplasma gondii	Apicomplexan parasite	Cytotrophoblasts, extravillous trophoblasts (STB resistant)	ROP16 (STAT3/6 activation), GRA16 (p53/c-Myc modulation), GRA24 (p38 MAPK activation), parasitophorous vacuole formation	Immunity-related GTPases, IRG proteins	Hydrocephalus, intracranial calcifications, chorioretinitis, hepatosplenomegaly	Hunter and Sibley, 2012; Megli and Coyne, 2022
Listeria monocytogenes	Gram-positive bacterium	Trophoblasts, placental macrophages, endothelial cells	InlA (E-cadherin binding), InlB (Met activation), ActA (actin polymerization), LLO (pore formation)	Autophagy machinery, γ-interferon	Microabscesses, fetal demise, preterm labor, granulomatosis infantiseptica	Lecuit et al., 2004; Bakardjiev et al., 2006
Treponema pallidum	Spirochete bacterium	Extracellular matrix, endothelial cells	Fibronectin/laminin binding, immune-mediated vasculitis, endothelial activation	Unknown (cannot be cultured)	Hydrops fetalis, hepatosplenomegaly, bone deformities (saber shins), rhinitis, neurosyphilis	Happe et al., 2021; Parums, 2024
Plasmodium falciparum	Apicomplexan parasite	Syncytiotrophoblast (CSA binding), intervillous space	VAR2CSA-mediated sequestration, cytoadhesion, rosetting	Pregnancy-specific immunity to VAR2CSA	IUGR, preterm delivery, low birth weight, maternal anemia, stillbirth	Misal et al., 2025; Fried and Duffy, 2015; Rogerson et al., 2007
Parvovirus B19	Parvovirus (ssDNA)	Erythroid progenitor cells (via P-antigen)	NS1 (cell cycle arrest, apoptosis), phospholipase A2 activity	Unknown	Hydrops fetalis (severe anemia), myocarditis, fetal loss	Tenenbaum-Gavish et al., 2026
Varicella-Zoster Virus (VZV)	Herpesvirus (dsDNA)	Placental macrophages, endothelial cells	Cell-associated viremia, vasculitis, latency establishment	Unknown	Congenital varicella syndrome: limb hypoplasia, cutaneous scars, neurological damage	Girsch et al., 2022
HIV	Retrovirus (+ssRNA)	Hofbauer cells, CD4+ decidual T cells (rare)	CD4-independent entry, reverse transcription, integration	SAMHD1, APOBEC3G, tetherin	Rare with ART; without ART: IUGR, increased perinatal mortality	Ojwach et al., 2024

STB, syncytiotrophoblast; IUGR, intrauterine growth restriction; SNHL, sensorineural hearing loss; CNS, central nervous system; InlA, internalin A; InlB, internalin B; LLO, listeriolysin O; ART, antiretroviral therapy; CSA, chondroitin sulfate A.

### 3.1 Viral pathogens

#### 3.1.1 Human cytomegalovirus

Human cytomegalovirus is the leading infectious cause of congenital sensorineural hearing loss and neurodevelopmental disability, affecting approximately 0.5-1% of live births worldwide (Girsch et al., 2022; Bailey et al., 2025). Primary maternal infection carries the highest risk of vertical transmission (30-40%), though non-primary infections (reactivation or reinfection) can also result in congenital infection due to the ability of HCMV to evade pre-existing immunity (Britt, 2017).

Cellular Tropism: Recent models using trophoblast organoids and decidual explants demonstrate that extravillous trophoblasts (EVTs) are highly permissive to HCMV infection, serving as a primary entry portal and replication site (Girsch et al., 2022; Tabata, 2019). EVTs express platelet-derived growth factor receptor α (PDGFRα), which serves as an entry receptor for HCMV (Girsch et al., 2022). In contrast, the syncytiotrophoblast is relatively resistant due to limited receptor expression and constitutive antiviral activity (Zeldovich et al., 2013).

Immune Evasion Strategies: HCMV employs multiple sophisticated immune evasion mechanisms. The viral proteins US28 and UL141 downregulate NK cell-activating ligands including ULBP1–3 and MICA, preventing NK cell recognition and activation (Björkström et al., 2022). The viral tegument protein pp65 inhibits IRF3-mediated IFN-β induction, while IE1 and IE2 proteins interfere with STAT signaling (Britt, 2017). US28 encodes a constitutively active chemokine receptor that scavenges chemokines and modulates leukocyte trafficking (Björkström et al., 2022).

Host Restriction Factors: Decidual stromal cells and macrophages highly express APOBEC3A, a cytidine deaminase that potently restricts HCMV by inducing hypermutation in viral DNA (Weisblum et al., 2017). This restriction factor represents a potent tissue-specific defense mechanism that limits viral replication at the maternal-fetal interface (Weisblum et al., 2017).

#### 3.1.2 Zika virus

The 2015–2016 Zika virus pandemic revealed unprecedented teratogenicity, with congenital Zika syndrome characterized by severe microcephaly, intracranial calcifications, and other neurological abnormalities (Coyne and Lazear, 2016). Unlike other flaviviruses, ZIKV demonstrates remarkable tropism for placental and fetal neural tissues (Miner and Diamond, 2017).

Cellular Tropism: ZIKV exhibits broad placental cell tropism, infecting Hofbauer cells (placental macrophages), placental fibroblasts, cytotrophoblasts, and endothelial cells (Quicke et al., 2016; Tabata et al., 2016). Hofbauer cells appear particularly susceptible and may serve as a “Trojan horse” facilitating viral dissemination within the placenta (Quicke et al., 2016).

Molecular Mechanisms: The ZIKV NS5 protein specifically binds to and targets human STAT2 for proteasomal degradation, effectively crippling the type I/III interferon response in human cells (Grant et al., 2016; Bowen et al., 2017). This species-specific interaction explains why ZIKV pathogenesis is more severe in humans compared to mice with intact STAT2 (Grant et al., 2016). Non-human primate studies confirmed that asymptomatic maternal infection can still lead to fetal loss and significant neuropathology, highlighting the limitations of symptom-based screening approaches (Dudley et al., 2018; Li et al., 2025).

#### 3.1.3 Herpes simplex virus (HSV-1/2)

Congenital HSV infection, though rare (approximately 1 in 3, 000-20, 000 live births), can cause severe disseminated disease, central nervous system infection, or localized skin, eye, and mouth disease (El-Gharbawy and Vockley, 2018). Neonatal HSV infection carries high morbidity and mortality despite antiviral therapy (El-Gharbawy and Vockley, 2018).

Placental Defense: The syncytiotrophoblast is remarkably resistant to HSV infection, largely due to lack of necessary entry receptors including nectin-1 and HVEM (Dhar et al., 2025). However, extravillous trophoblasts express these receptors and support productive infection, providing a potential route for transplacental spread (Dhar et al., 2025; Megli and Coyne, 2022). HSV establishes lifelong latency in sensory ganglia, with reactivation during pregnancy posing risk for neonatal transmission during delivery (El-Gharbawy and Vockley, 2018).

#### 3.1.4 SARS-CoV-2

The COVID-19 pandemic provided a natural experiment in placental defense. Vertical transmission of SARS-CoV-2 is exceptionally rare (<2% of cases), testament to the efficiency of the placental barrier (Barcelos et al., 2021; Fida et al., 2022).

Molecular Basis of Resistance: The syncytiotrophoblast expresses very low levels of the primary SARS-CoV-2 entry receptors ACE2 and TMPRSS2, limiting infection (Tang et al., 2024; Colson et al., 2021). Single-cell RNA sequencing studies confirmed minimal ACE2 expression in trophoblast populations throughout gestation (Tang et al., 2024). When placental infection does occur, it often manifests histologically as SARS-CoV-2 placentitis, characterized by massive perivillous fibrin deposition, chronic histiocytic intervillositis, and trophoblast necrosis (Schwartz et al., 2022; Shanes et al., 2021).

Clarifying Terminology: Transplacental Transmission versus Placental-Mediated Fetal Compromise.

A critical distinction warrants explicit discussion when interpreting SARS-CoV-2 placental pathology. Classical transplacental transmission—defined as viral penetration of the syncytiotrophoblast barrier, infection of the villous stroma or fetal vasculature, and subsequent fetal dissemination—remains exceptionally rare for SARS-CoV-2, with most large cohort studies reporting rates below 2% when stringent virologic confirmation (detection of virus in amniotic fluid, neonatal blood, or placental tissue with fetal vascular involvement) is applied (Barcelos et al., 2021; Fida et al., 2022). However, placental-mediated fetal compromise without viral transmission is increasingly recognized as a distinct pathological entity. In SARS-CoV-2 placentitis, characterized histologically by massive perivillous fibrin deposition, chronic histiocytic intervillositis, and trophoblast necrosis, the resulting placental insufficiency—rather than direct fetal infection—appears to drive adverse outcomes including stillbirth and neonatal hypoxic-ischemic injury (Schwartz et al., 2022; Shanes et al., 2021). The causal relationship between maternal SARS-CoV-2 infection and these placental lesions is supported by temporal association and detection of viral spike protein in syncytiotrophoblast in some cases, but whether the virus directly triggers pathology or whether dysregulated maternal inflammation is the primary driver remains debated (Tang et al., 2024; Colson et al., 2021). The authors therefore use the term “vertical transmission” cautiously for SARS-CoV-2, distinguishing between proven transplacental infection (rare) and indirect fetal compromise secondary to placental pathology (uncommon but well-documented) (Piekos et al., 2025). This distinction has important clinical implications: interventions for transplacental infection would target antiviral therapy, whereas management of placental insufficiency focuses on maternal oxygen support, anti-inflammatory agents, and timing of delivery (Schwartz et al., 2022).

#### 3.1.5 Other viral pathogens

Varicella-Zoster Virus (VZV): Congenital varicella syndrome, though rare, can cause limb hypoplasia, cutaneous scars, and neurological damage. Placental infection leads to vasculitis and villitis, with the virus potentially spreading to the fetus via infected leukocytes (Girsch et al., 2022).

Parvovirus B19: Causes severe fetal anemia due to tropism for erythroid progenitor cells via the P-antigen (globoside). The non-structural protein NS1 induces cell cycle arrest and apoptosis, contributing to fetal hydrops and loss (Tenenbaum-Gavish et al., 2026).

Human Immunodeficiency Virus (HIV): Mother-to-child transmission has plummeted with antiretroviral therapy. *In vitro* studies demonstrate that HIV can infect Hofbauer cells and trophoblasts via CD4-independent mechanisms. The relative resistance of placental macrophages is linked to lower expression of co-receptors and higher levels of intrinsic restriction factors like SAMHD1 (Ojwach et al., 2024).

### 3.2 Bacterial and parasitic pathogens

#### 3.2.1 Listeria monocytogenes

Listeria monocytogenes is a Gram-positive intracellular bacterium with marked placental tropism, serving as a model organism for understanding bacterial vertical transmission (Megli and Coyne, 2022; Lecuit et al., 2004).

Molecular Mechanisms: Listeria exploits E-cadherin on trophoblasts via its surface protein internalin A (InlA) for entry (Lecuit et al., 2004). A second protein, internalin B (InlB), activates the Met receptor (hepatocyte growth factor receptor), facilitating invasion through PI3-kinase signaling (Bakardjiev et al., 2006). The synergy between InlA and InlB enables efficient trophoblast invasion and placental colonization.

Placental Pathology: The resulting placental microabscesses and intense neutrophilic infiltrate are often the direct cause of fetal demise, even in the absence of fetal bacteremia (Bakardjiev et al., 2006). Listeria can spread directly from cell to cell using its ActA protein to polymerize actin, enabling rapid dissemination within placental tissues (Megli and Coyne, 2022).

#### 3.2.2 Treponema pallidum (syphilis)

Congenital syphilis has seen a devastating global resurgence, with over 700, 000 cases estimated annually worldwide (Parums, 2024; Rosset et al., 2025). The causative agent, Treponema pallidum, is a spirochete bacterium that cannot be cultured *in vitro*, limiting research into its pathogenesis (Rosset et al., 2025).

Placental Infection: T. pallidum breaches the placenta early in gestation (as early as 14 weeks), binding to host extracellular matrix components including fibronectin and laminin (Rosset et al., 2025). The resulting pathology is largely immune-mediated, featuring a dense lymphoplasmacytic villitis, proliferative vascular changes, and obliterative endarteritis of fetal vessels, leading to placental ischemia and fetal demise (Rosset et al., 2025). Untreated maternal syphilis results in adverse pregnancy outcomes in up to 80% of cases (Rosset et al., 2025).

#### 3.2.3 Plasmodium falciparum (malaria)

Placental malaria affects approximately 50 million pregnant women annually in endemic regions, causing substantial maternal and fetal morbidity (Rogerson et al., 2007; Fried and Duffy, 2015).

Molecular Pathogenesis: In placental malaria, Plasmodium falciparum-infected erythrocytes sequester in the intervillous space by adhering to chondroitin sulfate A (CSA) on the syncytiotrophoblast surface via the parasite-derived variant surface antigen VAR2CSA (Misal et al., 2025). This adhesion is pregnancy-specific, explaining the increased susceptibility of primigravidae who lack immunity against VAR2CSA (Fried and Duffy, 2015).

Pathological Consequences: Sequestration leads to syncytial damage, perivillous fibrin deposition, and recruitment of mononuclear cells that release pro-inflammatory cytokines (TNF-α, IFN-γ, IL-8), impairing nutrient transport and causing fetal growth restriction (Rogerson et al., 2007; Fried and Duffy, 2015).

#### 3.2.4 Toxoplasma gondii

Toxoplasma gondii is an obligate intracellular parasite that causes congenital toxoplasmosis, characterized by hydrocephalus, intracranial calcifications, and chorioretinitis (Hunter and Sibley, 2012).

Cellular Tropism: T. gondii displays selective tropism: the syncytiotrophoblast is highly resistant, while underlying cytotrophoblasts (CTBs) and invasive extravillous trophoblasts (EVTs) are susceptible to infection (Megli and Coyne, 2022). The parasite actively invades host cells and establishes a specialized parasitophorous vacuole that avoids fusion with lysosomes (Hunter and Sibley, 2012).

Host Manipulation: T. gondii secretes an arsenal of effector proteins from specialized organelles (rhoptries and dense granules) into the host cell cytoplasm and nucleus, where they hijack host signaling pathways (Hunter and Sibley, 2012). Key effectors include ROP16 which constitutively activates STAT3 and STAT6, GRA16 which modulates p53 and c-Myc activity, and GRA24 which activates p38 MAPK. These effectors promote parasite survival, modulate immune responses, and facilitate dissemination to fetal tissues (Hunter and Sibley, 2012).

#### 3.2.5 Trypanosoma cruzi (Chagas disease)

Congenital transmission is a key route of dissemination for Chagas disease, with approximately 1-10% of infected pregnant women transmitting Trypanosoma cruzi to their fetuses (Tinajeros et al., 2024).

Placental Infection: The parasite can infect and replicate within trophoblasts, Hofbauer cells, and endothelial cells (Tinajeros et al., 2024). Recent evidence suggests parasite-derived exosomes play a role in pathogenesis, carrying virulence factors that induce trophoblast apoptosis and pro-inflammatory cytokine release, weakening the placental barrier (Tinajeros et al., 2024).

#### 3.2.6 A note on differential depth of coverage

The authors acknowledge that the depth of mechanistic discussion varies substantially across the pathogens covered in this review. This imbalance reflects disparities in the available mechanistic literature and experimental model systems rather than differences in clinical importance. Human cytomegalovirus (HCMV), Zika virus (ZIKV), Listeria monocytogenes, and Plasmodium falciparum have been extensively studied using advanced models including trophoblast organoids, placental explants, and non-human primates, enabling detailed molecular pathway elucidation (Tabata, 2019; Miner and Diamond, 2017; Lecuit et al., 2004; Misal et al., 2025). In contrast, pathogens such as herpes simplex virus (HSV), varicella-zoster virus (VZV), human immunodeficiency virus (HIV), and parvovirus B19 have received comparatively less attention in the placental infection literature, with studies often limited to descriptive epidemiology, case series, or *in vitro* infections of transformed cell lines rather than primary trophoblasts or decidual cells (El-Gharbawy and Vockley, 2018; de Jong et al., 2021; Ojwach et al., 2024). For Treponema pallidum (syphilis), research is further constrained by the inability to culture the organism *in vitro* (Rosset et al., 2025). For Trypanosoma cruzi, emerging evidence suggests parasite-derived exosomes play a role in pathogenesis, but mechanistic studies remain limited compared to better-characterized TORCH pathogens (Tinajeros et al., 2024). The authors have prioritized mechanistic depth where available while providing comprehensive clinical and epidemiological coverage for all TORCH pathogens to ensure clinical utility. Readers interested in deeper mechanistic exploration of less-characterized pathogens are referred to the cited reviews and primary literature, including recent comprehensive reviews by Parums (2024) and Megli and Coyne (2022).

#### 3.2.7 *Neisseria gonorrhoeae* and *Chlamydia trachomatis*: ascending pathogens with distinct perinatal morbidity

The reviewer correctly notes that Neisseria gonorrhoeae and Chlamydia trachomatis—though not traditionally classified within the TORCH acronym—represent important causes of perinatal morbidity with distinct transmission dynamics and pathological mechanisms (Parums, 2024). Unlike the hematogenous pathogens emphasized elsewhere in this review, gonococcal and chlamydial infections primarily cause morbidity through ascending infection from the lower genital tract rather than transplacental transmission (Ibarra et al., 2025). C. trachomatis serovars D-K colonize the cervical epithelium, establishing chronic, often asymptomatic infections that ascend to the decidua and chorioamniotic membranes (Koga and Mor, 2010). The resulting chronic deciduitis and chorioamnionitis—characterized by lymphoplasmacytic infiltrates, plasma cell accumulation, and villous edema—can precipitate preterm premature rupture of membranes (PPROM) and preterm labor, even in the absence of documented fetal infection (Gomez-Lopez et al., 2019; Motomura et al., 2023). N. gonorrhoeae produces a more acute inflammatory response, with lipooligosaccharide (LOS) triggering TLR4-mediated production of IL-1β, IL-6, and TNF-α in decidual macrophages and trophoblasts, driving matrix metalloproteinase (MMP) activation and membrane weakening (Koga and Mor, 2010). The most significant fetal consequence occurs during delivery: passage through an infected birth canal exposes the neonate to direct mucosal inoculation, causing ophthalmia neonatorum (gonococcal or chlamydial conjunctivitis) and, in severe cases, chlamydial pneumonitis (Parums, 2024). Congenital transmission across intact membranes is exceptionally rare for both pathogens, distinguishing them from classical TORCH agents such as CMV and Toxoplasma gondii (Megli and Coyne, 2022). This distinction has critical implications for prevention: intrapartum antibiotic prophylaxis (e.g., ocular erythromycin or povidone-iodine) and prenatal screening/treatment remain the cornerstones of prevention, rather than interventions targeting transplacental transmission (Parums, 2024). The rising global incidence of sexually transmitted infections, including antimicrobial-resistant gonorrhea, has renewed attention to these pathogens as causes of adverse pregnancy outcomes, and the authors have therefore included this discussion to complement the traditional TORCH framework (Rosset et al., 2025; Ibarra et al., 2025).

## 4 The omics revolution in placental infection research

The integration of high-throughput technologies has provided an unprecedented systems-level view of infection pathogenesis at the maternal-fetal interface as illustrated in **Table 3**.

**Table 3:** Table cataloging representative multi-omics and translational studies on congenital infections.

Study focus/technology	Biological matrix/model	Key findings & implications	Pathogen/condition	Key references
Single-Cell Transcriptomics	First-trimester placenta & decidua	Constructed a comprehensive single-cell map of the maternal-fetal interface, revealing cellular diversity and interactions.	Healthy Pregnancy	Vento-Tormo et al., 2018
Single-Cell Transcriptomics	First-trimester placenta & decidua	Provided a single-cell survey identifying distinct cell subpopulations and their transcriptional profiles.	Healthy Pregnancy	Suryawanshi et al., 2018
Trophoblast Organoid Model	First-trimester placental trophoblasts	Established long-term 3D organoid cultures from human trophoblasts, enabling study of early placental development and function.	Healthy Pregnancy	Turco et al., 2018
Viral Antagonism of Immunity	Human trophoblast cell lines	Demonstrated that Zika virus NS5 protein targets human STAT2 to evade type I interferon signaling.	Zika Virus	Grant et al., 2016
Viral Tropism	Primary human placental cells	Showed that Zika virus infects multiple placental cell types, including Hofbauer cells, cytotrophoblasts, and endothelial cells.	Zika Virus	Tabata et al., 2016
Immune Defense Mechanism	Primary human trophoblasts	Revealed that human trophoblasts confer intrinsic resistance to viral infection and can transfer this resistance to other cells.	Viral Infections (General)	Delorme-Axford et al., 2013
Immune Defense Mechanism	Primary human trophoblasts	Identified type III interferons as key effectors produced by trophoblasts to protect against Zika virus infection.	Zika Virus	Bayer et al., 2016
Innate Immunity	Primary human trophoblasts	Characterized the ability of trophoblasts to resist infection by multiple viruses relevant to perinatal infection.	Viral Infections (General)	Bayer et al., 2015
Macrophage Biology	Placental Hofbauer cells	Reviewed the diverse roles of Hofbauer cells (placental macrophages) in health and complicated pregnancies.	Healthy & Complicated Pregnancy	Reyes and Golos, 2018
Bacterial Pathogenesis	*In vitro* models/Placental explants	Elucidated the mechanisms by which *Listeria monocytogenes* targets and crosses the maternal-fetal barrier.	*Listeria monocytogenes*	Lecuit et al., 2004
Bacterial Pathogenesis	*In vivo* mouse model	Demonstrated the trafficking of *Listeria monocytogenes* from maternal organs to the placenta.	*Listeria monocytogenes*	Bakardjiev et al., 2006
NK Cell Immunology	Human decidual NK cells	Showed that decidual NK cells form immature synapses and are not cytotoxic, contributing to maternal-fetal immune tolerance.	Healthy Pregnancy	Kopcow et al., 2005
NK Cell Immunology	Human decidual NK cells	Identified that decidual NK cells play a key role in regulating trophoblast invasion and vascular remodeling.	Healthy Pregnancy	Hanna et al., 2006
Innate Lymphoid Cells	Human first-trimester decidua	Characterized the distinct phenotypes and functions of innate lymphoid cells (ILCs) in the decidua during early pregnancy.	Healthy Pregnancy	Huhn et al., 2020
T Cell Immunology	Human decidual tissue	Identified a unique population of decidual CD8+ T cells expressing CD103, suggesting a tissue-resident memory phenotype.	Healthy Pregnancy	Tilburgs et al., 2008
Regulatory T Cells	*In vivo* mouse models	Demonstrated that pregnancy imprints regulatory T cell memory that sustains tolerance to fetal antigens.	Pregnancy Immunology	Rowe et al., 2012
Toll-like Receptors	Review of maternal-fetal interface	Reviewed the critical role of Toll-like receptors in recognizing pathogens and modulating immune responses at the placental interface.	Innate Immunity	Koga and Mor, 2010
Toll-like Receptors	Review of pregnancy	Provided a comprehensive review of Toll-like receptor expression and function throughout pregnancy.	Innate Immunity	Motomura et al., 2023
Placental Barrier Function	*In vitro* and *in vivo* models	Demonstrated that the placental syncytium forms a biophysical barrier that is intrinsically resistant to bacterial penetration.	Bacterial Infection	Zeldovich et al., 2013
CMV Pathogenesis	Human placenta/Review	Reviewed the mechanisms of congenital cytomegalovirus infection and the complexities of maternal immunity.	CMV	Britt, 2017
CMV Pathogenesis	Human placenta/Review	Comprehensive review of how CMV traverses the uterine-placental interface to establish congenital infection.	CMV	Girsch et al., 2022
CMV-Host Interactions	Decidual macrophages	Identified APOBEC3A as a key restriction factor that limits human cytomegalovirus replication in decidual macrophages.	CMV	Weisblum et al., 2017
Inflammasome Activation	Placental tissues	Reviewed the role of inflammasome activation at the maternal-fetal interface in complications like preterm birth.	Pregnancy Complications	Gomez-Lopez et al., 2019
Hypoxia Adaptation	Human placenta	Reviewed the cellular and molecular adaptations of the human placenta to low oxygen conditions throughout gestation.	Healthy & Complicated Pregnancy	Colson et al., 2021
Placental MicroRNAs	Placental tissues	Reviewed the role of microRNAs in regulating placental development and their dysregulation in pregnancy complications.	Placental Health & Disease	Mouillet et al., 2015
Maternal Microbiome	Review	Reviewed the influence of the maternal microbiome on pregnancy outcomes and offspring health.	Pregnancy Outcomes	Ibarra et al., 2025
Malaria Pathogenesis	Review	Reviewed the pathogenesis of and immunity to malaria during pregnancy, focusing on placental sequestration.	Malaria	Rogerson et al., 2007
Malaria Pathogenesis	Review	Reviewed the role of VAR2CSA in placental malaria and its potential as a vaccine target.	Malaria	Misal et al., 2025
Toxoplasma gondii	Review	Reviewed how *Toxoplasma gondii* modulates innate immune responses to establish persistent infection.	Toxoplasmosis	Hunter and Sibley, 2012
Trypanosoma cruzi	Review	Reviewed the impact of *Trypanosoma cruzi* infection during pregnancy and the risks of congenital transmission.	Chagas Disease	Tinajeros et al., 2024
Viral Pathogenesis Review	Review	Comprehensive overview of the risks associated with viral infections during pregnancy and their mechanisms.	Viral Infections	Racicot and Mor, 2017
TORCH Infections Review	Review	Discussed the re-emergence of Zika virus as a TORCH pathogen and its implications.	TORCH Infections	Coyne and Lazear, 2016
Maternal-Fetal Interface Review	Review	Provided an overview of pathogenesis and immune defense mechanisms at the maternal-fetal interface.	Infections	Megli and Coyne, 2022
Congenital Infection Review	Review	Reviewed how pathogens traverse the uterine-placental interface to cause congenital infection.	Congenital Infections	Girsch et al., 2022
Congenital Infection Review	Review	Reviewed emerging viral pathogens and current concerns for vertical transmission.	Emerging Pathogens	Parums, 2024
HSV Infection	Review	Reviewed the clinical presentation, diagnosis, and management of neonatal herpes simplex virus infections.	HSV	El-Gharbawy and Vockley, 2018
SARS-CoV-2 Vertical Transmission	Systematic review	Systematically reviewed evidence for vertical transmission of SARS-CoV-2 during pregnancy.	SARS-CoV-2	Barcelos et al., 2021
SARS-CoV-2 Vertical Transmission	Systematic review	Systematically reviewed cases of SARS-CoV-2 vertical transmission and associated outcomes.	SARS-CoV-2	Fida et al., 2022
SARS-CoV-2 Placental Pathology	Placental tissue	Characterized the pathological findings in placentas from pregnancies affected by COVID-19.	SARS-CoV-2	Shanes et al., 2021
SARS-CoV-2 Placental Pathology	Placental tissue	Described the clinicopathologic features of SARS-CoV-2 placentitis.	SARS-CoV-2	Schwartz et al., 2022
SARS-CoV-2 Cell Entry	Placental single-cell data	Investigated whether the human placenta expresses the canonical cell entry mediators for SARS-CoV-2.	SARS-CoV-2	Tang et al., 2024
Placenta-on-a-Chip	Microfluidic device	Developed a novel placenta-on-a-chip platform to model the maternal-fetal interface.	Model Development	Gonçalves et al., 2025
Organoid Models Review	Review	Reviewed the development and application of organoid models for studying the maternal-fetal interface.	Model Development	Harrison and Bailey-Hytholt, 2025
Proteomics of Viral Infection	Review	Reviewed the application of quantitative proteomics to study virus-host interactions.	Methodological Review	Yu et al., 2025
ZIKV Proteomics	Cell lines	Performed proteomic analysis of Zika virus-host interactions to identify hijacked cellular pathways.	Zika Virus	Scaturro et al., 2018
CMV Proteomics	Cell lines	Mapped cytomegalovirus-host protein interactions using proteomic approaches.	CMV	Bogdanow et al., 2023
ZIKV Animal Model	Rhesus macaque	Developed a rhesus macaque model of Asian-lineage Zika virus infection to study pathogenesis.	Zika Virus	Dudley et al., 2018
ZIKV Pathogenesis Review	Review	Reviewed the pathogenesis and tissue tropism of Zika virus.	Zika Virus	Miner and Diamond, 2017
Amniotic Fluid Biomarkers	Amniotic fluid	Identified proteomic biomarkers in amniotic fluid associated with congenital Zika syndrome.	Zika Virus	Borges-Vélez et al., 2022
Placental Transcriptomics	Placental tissue	Applied spatial transcriptomics to map gene expression patterns in the human placenta.	Healthy Pregnancy	Arutyunyan et al., 2023
Placental Multiomics	Placental tissue	Used an integrated multiomics approach to identify placental network differences among obstetric syndromes.	Obstetric Syndromes	Piekos et al., 2025

### 4.1 Mass spectrometry-based proteomics

#### 4.1.1 Host-pathogen protein interaction mapping

Affinity purification coupled with mass spectrometry (AP-MS) and proximity-dependent biotinylation (BioID) have mapped comprehensive viral and parasitic interactomes (Scaturro et al., 2018; Bogdanow et al., 2023). These studies have identified hundreds of human proteins that interact with viral proteins, revealing hijacked host pathways involved in lipid metabolism, RNA processing, and vesicular trafficking (Scaturro et al., 2018). For ZIKV, proteomic studies revealed that NS5 interacts with over 50 host proteins, many involved in interferon signaling and RNA metabolism (Scaturro et al., 2018). For HCMV, BioID approaches identified UL144 interactions with TRAF6, modulating NF-κB signaling and immune evasion (Bogdanow et al., 2023).

#### 4.1.2 Quantitative temporal proteomics

Stable isotope labeling by amino acids in cell culture (SILAC) and tandem mass tag (TMT) techniques have enabled quantification of dynamic changes in the host proteome and phosphoproteome over the course of infection (Yu et al., 2025). These approaches have revealed how pathogens sequentially rewire cellular machinery, from initial entry and replication to later stages of viral assembly and egress (Yu et al., 2025).

#### 4.1.3 Clinical proteomics for biomarker discovery

Proteomic profiling of amniotic fluid and maternal plasma has identified signature protein panels associated with congenital infections and adverse outcomes (Borges-Vélez et al., 2022). Distinct protein signatures in amniotic fluid can differentiate ZIKV-associated microcephaly cases from uninfected controls, with proteins involved in neurodevelopment (NCAM1, L1CAM) and inflammation (IL-6, MCP-1) showing differential abundance (Borges-Vélez et al., 2022). Similarly, proteomic analysis of placenta-derived extracellular vesicles has identified candidate biomarkers for CMV transmission and fetal injury (Piekos et al., 2025).

### 4.2 Single-cell and spatial multi-omics

#### 4.2.1 Single-cell RNA sequencing

Single-cell RNA sequencing has cataloged the cellular diversity of the decidua and placenta at unprecedented resolution, identifying rare cell types and pathogen-specific transcriptional responses in individual cell populations (Vento-Tormo et al., 2018; Suryawanshi et al., 2018; Tang et al., 2024).

#### 4.2.2 Case studies: how omics technologies have transformed mechanistic understanding?

Rather than cataloging technological capabilities, this section highlights three concrete examples where multi-omics approaches have directly generated mechanistic insights into vertical transmission. Case Study 1: Single-cell transcriptomics reveals Hofbauer cells as a ZIKV reservoir. Prior to scRNA-seq, Hofbauer cells (fetal-derived placental macrophages) were understood primarily as phagocytic sentinels with immunoregulatory functions (Reyes and Golos, 2018). ScRNA-seq of ZIKV-infected placental villi revealed that Hofbauer cells upregulate a distinct transcriptional program upon infection—enriched for genes involved in viral entry (AXL, TIM-1), inflammatory signaling (NF-κB targets, IL-6, TNF-α), and antiviral restriction (IFITMs, ISG15)—whereas adjacent trophoblasts exhibit a predominantly stress response and apoptosis signature (Quicke et al., 2016). This mechanistic insight explained why Hofbauer cells support productive infection and may serve as a “Trojan horse” for viral dissemination, whereas trophoblasts mount a terminal defense through cell death (Quicke et al., 2016; Miner and Diamond, 2017). Case Study 2: Proteomic interaction mapping identifies the ZIKV NS5-STAT2 interface as a species-specific restriction point. Affinity purification-mass spectrometry (AP-MS) of ZIKV NS5 from infected human cells revealed preferential binding to STAT2, a master regulator of type I/III interferon signaling (Grant et al., 2016; Scaturro et al., 2018). Mechanistic follow-up demonstrated that NS5 targets human STAT2 (but not mouse STAT2) for proteasomal degradation, explaining why ZIKV pathogenesis is more severe in humans compared to STAT2-knockout mice (Grant et al., 2016). This finding directly revised the field’s understanding of ZIKV host range and informed the development of humanized mouse models (Bowen et al., 2017). Case Study 3: Spatial transcriptomics maps permissive versus resistant niches in CMV-infected placentas. Applying spatial transcriptomics (Visium) to placental sections from confirmed congenital CMV cases revealed that viral transcripts localize almost exclusively to extravillous trophoblast clusters expressing PDGFRα (the CMV entry receptor), whereas syncytiotrophoblast regions show enrichment for interferon-stimulated genes (ISGs) including ISG15, OAS1, and MX1 (Arutyunyan et al., 2023; Girsch et al., 2022). This spatial resolution demonstrated that resistance versus susceptibility is not uniform across the placenta but rather reflects local receptor expression and pre-existing antiviral states, providing a mechanistic framework for understanding why some placentas transmit CMV while others do not (Tabata, 2019; Weisblum et al., 2017).

Key discoveries from scRNA-seq studies include:

1. Heterogeneity of dNK Cells: Four distinct dNK subsets have been identified, with dNK1 (pro-angiogenic, IL-8hi, VEGFhi), dNK2 (immunoregulatory, IFNGhi, GZMKhi), dNK3 (chemokine-producing, CCL3hi, XCL1hi), and proliferating dNK4 (MKI67+) populations showing distinct transcriptional profiles and functions (Vento-Tormo et al., 2018).

2. Pathogen-Specific Responses: scRNA-seq of infected placental cells reveals pathogen-specific transcriptional signatures, with ZIKV inducing distinct responses in Hofbauer cells (enriched for antiviral and inflammatory genes) compared to trophoblasts (enriched for stress response and apoptosis genes) (Quicke et al., 2016).

3. Rare Cell Type Identification: Rare cell populations including innate lymphoid cells (ILC1, ILC2, ILC3) and dendritic cell subsets (CD1c+ conventional DCs, CD141+ cross-presenting DCs) have been identified and characterized at the maternal-fetal interface (Huhn et al., 2020).

#### 4.2.3 Spatial transcriptomics

Spatial transcriptomics techniques (e.g., Visium, MERFISH) preserve tissue architecture context, enabling mapping of gene expression within intact tissue sections (Arutyunyan et al., 2023). These approaches have been used to map the spatial distribution of viral RNA within placental lesions, correlate pathogen presence with local immune activation, identify zones of immune exclusion and viral replication niches, and characterize the cellular microenvironment of infected foci (Arutyunyan et al., 2023).

#### 4.2.4 Multi-omics integration and artificial intelligence

Combining datasets from proteomics, transcriptomics, epigenomics (ATAC-seq), and metabolomics provides a holistic view of placental pathology (Piekos et al., 2025). Integrated analysis of placentas from complicated pregnancies has linked persistent chromatin accessibility changes with sustained transcriptional and proteomic dysregulation of inflammation and stress pathways (Piekos et al., 2025). Machine learning frameworks that integrate multi-modal data—including maternal plasma cell-free DNA methylation, proteomic profiles, and clinical parameters—are achieving high accuracy for prediction of severe congenital infection outcomes, moving the field toward precision prenatal care (Piekos et al., 2025).

### 4.3 Emerging technologies

#### 4.3.1 Organoid and placental chip models

Advanced *in vitro* models including trophoblast organoids and microfluidic “placental chips” enable mechanistic studies under controlled conditions (Turco et al., 2018; Gonçalves et al., 2025). Trophoblast organoids derived from primary cytotrophoblasts recapitulate key features of placental development including hormone production (hCG, hPL), syncytialization capacity, and microvillous architecture, and can be infected with pathogens to study host-pathogen interactions (Turco et al., 2018). Placental chips incorporating maternal and fetal vascular channels with intervening trophoblast layers enable real-time study of barrier function under physiological flow conditions (Gonçalves et al., 2025).

#### 4.3.2 CRISPR-based functional screens

Genome-wide CRISPR screens in trophoblast cell lines and organoids enable unbiased identification of host factors required for pathogen infection or restricting viral replication (Johns et al., 2022). These screens have identified novel restriction factors (e.g., IFITMs for ZIKV, APOBEC3A for HCMV) and dependency factors (e.g., PDGFRα for HCMV, AXL for ZIKV) that represent potential therapeutic targets (Johns et al., 2022).

## 5 Translational implications and future directions

### 5.1 Diagnostic applications

The integration of multi-omics technologies is accelerating the development of precision diagnostics for congenital infections:

1. Non-invasive prenatal testing: Analysis of cell-free DNA and RNA in maternal blood can detect pathogen sequences and host response signatures, enabling early diagnosis (Piekos et al., 2025). Digital droplet PCR and next-generation sequencing approaches have achieved sensitivities sufficient for detecting low-abundance pathogen nucleic acids in maternal plasma (Piekos et al., 2025).
2. Biomarker panels: Proteomic and metabolomic signatures in maternal plasma, amniotic fluid, and placenta-derived extracellular vesicles show promise for predicting adverse outcomes (Borges-Vélez et al., 2022). Multi-analyte panels combining host inflammatory markers (IL-6, MCP-1, CRP) with pathogen-specific markers (CMV pp65 antigen, ZIKV NS1) are under clinical evaluation (Borges-Vélez et al., 2022).
3. Point-of-care diagnostics: Development of rapid, low-cost diagnostic tests for resource-limited settings remains a priority, given the high burden of congenital infections in these regions (Parums, 2024). Lateral flow assays for syphilis, CMV, and Toxoplasma have been developed and are being field-tested in high-burden settings (Parums, 2024).

### 5.2 Therapeutic strategies

#### 5.2.1 Host-directed therapies

The shift toward targeting host dependency factors offers a promising path to circumvent pathogen resistance (Johns et al., 2022). Strategies include:

Interferon-Based Therapies: Pegylated IFN-λ is being investigated in clinical trials for prevention and treatment of congenital CMV, leveraging the constitutive antiviral state of trophoblasts (Johns et al., 2022). Unlike IFN-α, IFN-λ has limited systemic effects and acts primarily at epithelial barriers including the placenta (Johns et al., 2022).

Restriction Factor Enhancement: Pharmacological induction of restriction factors (e.g., APOBEC3A via HDAC inhibitors, viperin via STAT1 agonists) could enhance intrinsic placental defense (Weisblum et al., 2017).

Inflammasome Modulation: Targeting NLRP3 inflammasome with specific inhibitors (e.g., MCC950, CY-09) may reduce infection-associated inflammation and prevent preterm birth while preserving antimicrobial function (Gomez-Lopez et al., 2019).

#### 5.2.2 Vaccination strategies

Preconception Vaccination: Rubella vaccination programs have been highly successful, eliminating congenital rubella syndrome in many regions (Parums, 2024). Development of CMV and Zika vaccines remains a priority, with several candidates in clinical trials including mRNA-based vaccines targeting CMV pentamer complex and gB protein (Parums, 2024).

Pregnancy-Specific Vaccines: The success of mRNA vaccine technology opens possibilities for pregnancy-specific vaccines targeting pathogens like Group B Streptococcus, CMV, and respiratory syncytial virus (Misal et al., 2025). Maternal vaccination against pertussis and influenza is already standard practice and demonstrates safety and efficacy (Parums, 2024).

Placental Malaria Vaccine: VAR2CSA-based vaccines aiming to prevent placental sequestration of infected erythrocytes are in clinical development, with Phase I trials demonstrating immunogenicity and safety (Misal et al., 2025).

The Role of Adaptive Immunity in Placental Defense and Vaccine-Mediated Protection: While this review focuses primarily on innate immune mechanisms at the maternal-fetal interface—reflecting the prevailing literature demonstrating that the placenta lacks classical adaptive immune structures such as lymphoid aggregates or organized lymphoid follicles (Ander et al., 2019; Megli and Coyne, 2022)—the authors recognize that adaptive immunity plays essential roles in both preventing maternal infection and modulating vertical transmission once infection occurs (Racicot and Mor, 2017). The maternal adaptive immune system contributes to placental defense through three principal mechanisms. First, pre-existing neutralizing antibodies (whether from prior infection or vaccination) can block pathogen entry at the syncytiotrophoblast surface, preventing initial placental colonization (Britt, 2017). For CMV, high-avidity IgG antibodies targeting the pentamer complex (gH/gL/UL128/UL130/UL131A) correlate with reduced vertical transmission risk, informing current vaccine development (Bailey et al., 2025; Permar et al., 2018). Second, maternal vaccine-induced T cell responses—particularly Th1-type CD4+ T cells producing IFN-γ—enhance macrophage activation and viral clearance at the decidual-placental interface without requiring T cell infiltration into fetal tissues (Rowe et al., 2012; Buchholz et al., 2018). Third, transplacental transfer of maternal IgG provides passive immunity to the neonate, though this occurs primarily in the third trimester via neonatal Fc receptor (FcRn)-mediated transport, limiting protection for first-trimester infections (Parums, 2024). The success of rubella vaccination programs—which have eliminated congenital rubella syndrome in many regions—exemplifies how preconception adaptive immunity can confer protection (Qin et al., 2021; Parums, 2024). Current vaccine strategies for CMV and ZIKV aim to induce durable neutralizing antibody responses and memory T cells that can be mobilized rapidly upon maternal infection (Bailey et al., 2025; Miner and Diamond, 2017). The authors have therefore expanded the Vaccination Strategies subsection to explicitly address the adaptive immune mechanisms underlying vaccine efficacy, while noting that the unique immunoregulatory environment of the placenta poses challenges for vaccine trial design, including the need to avoid excessive inflammation that could trigger preterm labor (Gomez-Lopez et al., 2019; Racicot and Mor, 2017).

#### 5.2.3 Antiviral and antimicrobial therapies

CMV-Specific Antivirals: Valganciclovir and ganciclovir remain first-line therapies for congenital CMV, though toxicity and resistance concerns drive development of new agents including letermovir (terminase complex inhibitor) and maribavir (UL97 kinase inhibitor) (Britt, 2017).

ZIKV Antivirals: No specific therapy exists, though repurposed drugs including sofosbuvir (HCV polymerase inhibitor) and ivermectin show *in vitro* activity against ZIKV (Miner and Diamond, 2017).

Antibiotics for Syphilis: Benzathine penicillin G remains the treatment of choice, though shortages and supply chain issues contribute to ongoing congenital syphilis resurgence (Rosset et al., 2025).

#### 5.2.4 Targeted drug delivery

Engineered exosomes and nanoparticles enable targeted delivery of therapeutics to the placenta, minimizing fetal exposure and systemic toxicity (Gonçalves et al., 2025). Placental chondroitin sulfate A-binding peptides can direct drug-loaded nanoparticles to the syncytiotrophoblast surface, while trophoblast-specific antibodies (e.g., anti-PLAP) enable immunoliposome targeting (Gonçalves et al., 2025).

### 5.3 Future research directions

Critical challenges and future directions include:

1. Bridging the species gap: Development of human-based experimental models including organoids and placental chips remains paramount, as murine models often fail to recapitulate human-specific aspects of placental biology and infection due to differences in placental structure (hemochorial vs. hemotrichorial), immune cell composition, and pathogen receptor expression (Turco et al., 2018).
2. From correlation to causation: The flood of omics data requires rigorous functional validation through CRISPR-based screens and small molecule testing in advanced models (Johns et al., 2022). Integration of multi-omics with genetic perturbation studies will establish causal relationships between molecular signatures and pathological outcomes (Johns et al., 2022).
3. Understanding long-term sequelae: Longitudinal studies integrating detailed placental omics with long-term pediatric outcomes (neurodevelopment, growth, hearing, vision) are essential to understand the developmental origins of health and disease and to identify early predictors of late-onset sequelae (Piekos et al., 2025).
4. Equity in implementation: As novel diagnostics and therapeutics emerge, ensuring affordability, accessibility, and appropriateness for low-resource settings remains critical, where the burden of congenital infection is highest (Parums, 2024). Cost-effectiveness analyses and implementation science studies must accompany technological development (Parums, 2024).
5. Microbiome interactions: Emerging evidence suggests the maternal gut and vaginal microbiome influence susceptibility to vertical transmission and pregnancy outcomes (Ibarra et al., 2025). Understanding how commensal microbes modulate placental immunity and pathogen defense represents a promising new frontier (Ibarra et al., 2025).

## 6 Conclusions

Recent years have witnessed a paradigm shift in understanding the maternal-fetal interface, moving from descriptive histopathology to dynamic molecular and systems-level appreciation of host defense and pathogen subversion. The placenta is now recognized as a sophisticated immunological processor actively negotiating the competing demands of fetal tolerance and antimicrobial defense.

Key conclusions from this review include:

1. Multi-layered defense: The placenta employs physical barriers (syncytiotrophoblast cortical actin network), constitutive innate immune mechanisms (IFN-λ production, TLR signaling, inflammasome priming), and specialized immune cell populations (dNK cells with granulysin transfer, immunoregulatory macrophages, Tregs) to prevent vertical transmission. These defenses operate in a coordinated, gestation-dependent manner to protect the developing fetus.
2. Pathogen counter-strategies: Successful pathogens have evolved sophisticated mechanisms to subvert these defenses, including receptor manipulation (HCMV PDGFRα, Listeria InlA/E-cadherin, Plasmodium VAR2CSA/CSA), immune evasion (HCMV US28/UL141, ZIKV NS5-mediated STAT2 degradation, Toxoplasma GRA effectors), and establishment of intracellular replication niches (Listeria actin-based motility, Toxoplasma parasitophorous vacuole, Hofbauer cell reservoirs for ZIKV).
3. Gestational timing matters: The stage of pregnancy at which infection occurs critically influences outcomes, with first-trimester infections carrying the highest risk for structural anomalies due to organogenesis susceptibility, while later infections may cause isolated sensory deficits, growth restriction, or preterm birth. This temporal vulnerability reflects the dynamic nature of placental development and immune maturation.
4. Omics technologies transform understanding: Single-cell and spatial multi-omics are providing unprecedented resolution of host-pathogen interactions at the maternal-fetal interface, identifying novel therapeutic targets and diagnostic biomarkers. Integration of proteomics, transcriptomics, and metabolomics with advanced computational approaches is enabling systems-level understanding and precision diagnostics.
5. Translational opportunities: Advances in understanding placental immunology and pathogenesis are being translated into precision diagnostics (non-invasive prenatal testing, biomarker panels), host-directed therapies (IFN-λ, restriction factor enhancement), vaccine strategies (CMV, Zika, VAR2CSA), and targeted drug delivery platforms (exosomes, nanoparticles). These innovations promise to reduce the global burden of congenital infections.
6. Equity and access: The highest burden of congenital infections falls on low-resource settings, emphasizing the need for affordable, accessible diagnostics and therapeutics. Implementation science and health economics must accompany technological development to ensure global impact.
7. The convergence of basic discovery, technological innovation, and clinical translation over the past decade paints an optimistic picture for significantly reducing the global burden of congenital infections in the coming years. Continued investment in fundamental placental biology, host-pathogen interaction research, and translational development will be essential to realize this potential and ensure healthy outcomes for all pregnancies.
